# The Future of Graduate Instruction in Medicine

**Published:** 1910-04-09

**Authors:** 


### The Future of Graduate Instruction in Medicine.

Elsewhere in this issue we publish the interesting address delivered by Professor Robert Kutner, the well-known Director of the Kaiserin Friedrich Haus, at Berlin, on the occasion of the foundation of the International Committee for Medical Extension Courses at the Budapest Conference last year. In a following number we hope to give full details of the work that is being done by this committee. We cordially commend the reading of these articles to everyone interested in Post-Graduate Instruction in Medicine. Dr. Kutner's address, giving as it does a summary of what is being done in the various European countries where post-graduate instruction has been considered during the last ten years, must inevitably stir the English reader to activity in the matter. For against the other countries England's record in this matter is a poor one. Compare, for example, the brilliant advance that has been made in Germany during the last five years with the spasmodic and half-hearted efforts which have resulted in the establishment of our various post-graduate schools. Compare, again, the teaching in those latter schools with that given in the German classes, and, finally, contrast the results of the gratuitous system of instruction of which the German, Italian, Russian, and Dutch practitioner can avail himself, and those obtained here, where attendance at graduate instruction classes means an expenditure of time and money which few practitioners can afford. The factors that have hitherto retarded the development of graduate instruction in this country are various, and can scarcely be adequately summarised. One of the most potent is perhaps the want of system that prevails with us, and the poor quality of the teaching. It is a common fallacy to suppose that because a man is a good teacher at a medical school he is necessarily also a good graduate instructor. In the majority of cases the student teacher is a failure in graduate classes, and equally much a failure is the specialist, who has never been in private practice, and who has grossly exaggerated ideas of the importance of his own specialty—and therefore incidentally of himself as a blessed exponent of the creed. Another important factor is undoubtedly the question of expense. Our graduate classes are too expensive, especially those in which practical work is likely to be done. What is more, they are not within reach of the average country practitioner, or even, in the case of London, of the suburban doctor. The Orpington practitioner, for instance, who attends a class at the Polyclinic or the West London does so at a much greater expense of time and money than, say, the practitioner who lives at Treptow and attends a demonstration at the Charité. The self-sacrifice demanded in the former case is excessive, under present conditions almost prohibitive, while in the latter it is not too great for one who is really desirous of furthering his knowledge. These are merely points that occur to us at the moment. In a future issue we hope to deal at length with the whole question of graduate study in this country.

